# Reliability and validity of the Norwegian version of the Disabilities of the Arm, Shoulder and Hand questionnaire in patients with shoulder impingement syndrome

**DOI:** 10.1186/1471-2474-15-78

**Published:** 2014-03-12

**Authors:** Benjamin Haldorsen, Ida Svege, Yngve Roe, Astrid Bergland

**Affiliations:** 1 Department of Physiotherapy, Martina Hansens Hospital, Pb 823, 1346 Sandvika, Bærum, Norway; 2 Norwegian Research Center for Active Rehabilitation (NAR), Department of Orthopaedics, Oslo University Hospital, Oslo, Norway; 3 Department of Physiotherapy, Faculty of Health Sciences, Oslo and Akershus University College of Applied Sciences, Pb 4, St. Olavs plass, 0130 Oslo, Norway

**Keywords:** Shoulder impingement syndrome, Outcome measure, DASH, Test-retest reliability, Internal consistency, Construct validity

## Abstract

**Background:**

Patient-rated outcome measures (PROMs) are an important part of clinical decision-making in rehabilitation of patients with shoulder pain. The Disabilities of Arm, Shoulder and Hand (DASH) questionnaire was designed to measure physical disability and symptoms in patients with musculoskeletal disorders of the upper extremity and is one the most commonly used outcome measures for patients with shoulder pain. The purpose of this study was to investigate the reliability and validity of the Norwegian version of the DASH in patients with shoulder impingement syndrome.

**Methods:**

Sixty-three patients diagnosed with shoulder impingement syndrome at an orthopaedic outpatient clinic were included in the study. Internal consistency of the DASH was evaluated by the Cronbach’s alpha and item-to-total correlations. Test-retest reliability was analyzed by the intraclass correlation coefficient (ICC) and limits of agreement (LoA) according to the Bland Altman method. Standard error of measurement (SEM) and minimally detectable change (MDC) were calculated for the total DASH score. Construct validity was evaluated by testing six a priori hypotheses for the Pearson’s correlation coefficient between the DASH and the Shoulder Pain and Disability Index (SPADI), the 36-item Short Form Health Survey (SF-36) and a Numeric Pain Rating Scale (NPRS).

**Results:**

*Reliability:* Cronbach’s alpha of the DASH was 0.93 and item-to-total correlations ranged from 0.36 to 0.81. ICC was 0.89. The 95 percent LoA was calculated to be between -11.9 and 14.1. SEM was 4.7 and MDC 13.1. *Construct validity:* Eighty-three percent of the a priori hypotheses of correlation were confirmed. The DASH showed a high positive correlation of 0.75 with the SPADI, a negative moderate correlation of -0.48 to -0.62 with physical functioning, bodily pain and physical component summary of the SF-36 and a moderate positive correlation of 0.58 with the NPRS. DASH correlated higher with the physical component summary than with the mental component summary of the SF-36.

**Conclusions:**

The Norwegian version of the DASH is a reliable and valid outcome measure for patients with shoulder impingement syndrome.

## Background

Patient-rated outcome measures (PROMs) are an important part of clinical decision making in rehabilitation of patients with shoulder pain. A number of PROMs are available [1,2], of which the Disabilities of the Arm, Shoulder and Hand (DASH) outcome measure is one of the most commonly used. The DASH is a condition-specific PROM developed to assess physical disability and symptoms in people with musculoskeletal disorders of the upper extremity [3-5]. The original English version of the DASH has been translated and adapted into many languages [5,6]. It has shown to be reliable, valid and responsive in patients with shoulder pathologies [1,4,7].

Shoulder impingement syndrome is the most common clinical diagnosis for patients with shoulder pain [8,9] and can be defined as a symptomatic compression of the subacromial structures during elevation of the arm [10,11]. The main symptom is anterior-lateral shoulder pain when lifting the arm above shoulder level and during overhead activities. Contributing factors to the development of shoulder impingement syndrome include inflammation of the tendons and bursa, degeneration of the tendons, postural dysfunctions and weak or dysfunctional rotator cuff and scapular musculature [10,11]. Various terms are used for the same condition such as rotator cuff disease/tendinopathy, subacromial impingement syndrome and painful arc syndrome.

Measurement properties of an outcome measure are related to the population and context in which it is used. Before a translated outcome measure can be used with confidence in clinical or research settings, the measurement properties of the translated version need to be determined [12]. The measurement properties of the Norwegian language version of the DASH have not previously been investigated in non-rheumatic patients with shoulder pain. The objective of this study was to examine the reliability and construct validity of the DASH in patients with shoulder impingement syndrome.

## Methods

### Patients

The patients were recruited from an outpatient clinic from December 2007 to October 2010. We included adult patients with the primary diagnosis of shoulder impingement syndrome (M75.4 in the ICD-10). The patients were diagnosed by an orthopaedic surgeon and screened for inclusion in the study by a physiotherapist. The impingement diagnosis was based on reported symptoms and clinical findings such as anterior-lateral shoulder pain worsening during elevation of the arm and overhead activities, normal or close to normal passive range of motion of the shoulder and positive impingement sign [13].

Patients were excluded from the study if they had generalized pain, symptoms of cervical spine disease, had undergone surgery in the affected shoulder within the last six months or were unable to understand written and spoken Norwegian. In addition, patients were excluded if they had been diagnosed with any rheumatologic illness, chronic systemic disease or cardiac disease.

### Measures

#### The Disabilities of the Arm, Shoulder and Hand questionnaire (DASH)

The DASH was developed by the Institute for Work and Health and the American Academy of Orthopaedic Surgeons (AAOS) [3-5]. It was designed to be a discriminative and evaluative measure of physical disability and symptoms in patients with musculoskeletal disorders of the upper extremity, assessing the condition of the patient during the past week. It measures whether the respondent has the capacity to do an activity, regardless of how it is performed. The main part of the questionnaire, the DASH disability/symptoms score, contains 30 items: 21 items about the ability to perform certain physical activities, 5 items about the severity of pain, activity-related pain, tingling, weakness and stiffness and 4 items concerning the effect of the upper extremity problem on social activities, work, sleep and self-image. Each item is scored on a five-point ordinal scale. To calculate the DASH score all completed responses are summed and averaged. This value is subtracted by one and multiplied by 25, giving a total score ranging from best to worst on a 0–100 scale. At least 27 of the 30 items must be completed to calculate a score. The translation and adaptation process of the Norwegian language version of the DASH is described by Finsen (in Norwegian) [14].

#### The Shoulder Pain and Disability Index (SPADI)

The SPADI was designed to measure pain and disability in patients with shoulder disorders [15]. A Norwegian version is available [16]. It is a 13-item PROM divided into two subscales: the five-item pain subscale and the eight-item disability subscale. Each item in the original version is scored on a visual analogue scale from from 0 (no pain/no difficulty) to 11 (worst pain imaginable/ so difficult required help). The pain and disability scores are equally weighted and added for the total SPADI score, ranging from best to worst on a 0–100 scale. The Norwegian version of the SPADI has shown to have acceptable reliability and validity in patients with rotator cuff disease [17].

#### The Short Form 36 Health Survey (SF-36)

The 36 item Short Form Health Survey is a generic PROM developed to assess eight health domains [18]: physical functioning (PF), role-physical (RP), bodily pain (BP), general health (GH), vitality (VT), social functioning (SF), role-emotional (RE) and mental health (MH). Each of the eight domains is scored worst to best on a 0–100 scale. Two summary scores representing physical (Physical component summary; PCS) and mental health (Mental component summary; MCS) can be calculated. We used the Norwegian version 1.2 [19]. The measurement properties of the SF-36 have been tested extensively [18].

#### Numeric Pain Rating Scale (NPRS)

The patients were asked to rate their pain on average over the last week prior to assessment using an 11-point Numeric Pain Rating Scale from 0 (no pain) - 10 (worst possible pain) [20]. The NPRS is a commonly used outcome measure for patients with shoulder pain and has shown to be reliable and valid [21].

### Procedures

Each patient was scheduled for two visits approximately one week apart. At the first visit the patients filled out the DASH, SPADI, SF-36 and Numeric Pain Rating Scale. Descriptive data such as age, sex, symptom duration and employment status were also collected. At the second visit the patients filled out the DASH and SPADI and they were asked if the shoulder condition had changed since the first visit. The patients did not receive any treatment between the first and the second visit. The study protocol was approved by the Regional committees for medical and health research ethics (REC South East), and was carried out in accordance with the Helsinki Declaration. We obtained written informed consent from all the participants at inclusion.

### Statistical analysis

QualityMetric Health Outcomes Scoring Software 3.0 (QualityMetric Inc, Lincoln, Rhode Island, USA) was used to manage missing SF-36 data and to calculate the SF-36 scores. PAWS Statistics 18.0 for Windows (SPSS Inc, Chicago, Illinois, USA) and the Stata/IC 11.2 for Mac (StataCorp, College Station, Texas, USA) were used for the other analysis. Floor and ceiling effects were evaluated using histograms, and were considered to be present if more than 15% of the respondents achieved the lowest or highest possible score, respectively [22]. P-values less than 0.05 were considered statistically significant.

#### Reliability

Internal consistency was assessed using Cronbach’s alpha and item-to-total correlation coefficient. A Cronbach’s alpha between 0.70 and 0.95 was considered to indicate a good internal consistency [22]. Item-to-total correlations above 0.3 were considered good [23]. Test-retest reliability was calculated with the use of intraclass correlation coefficient (ICC); 2.1-two-way random effect model single measures. The ICC classifications of Fleiss were used to interpret the ICC values [24]: ICCs above 0.75 may indicate excellent reliability, values between 0.40 and 0.75 fair to good reliability and values below 0.40 poor reliability. All patients who reported their shoulder pain as unchanged between the first and the second visit were included in the test-retest reliability analysis. Measurement error was assessed by estimating the standard error of measurement (SEM), minimally detectable change (MDC) and limits of agreement (LoA). SEM was calculated as the square root of the within-subject total variance of an ANOVA analysis [25]. The MDC was calculated as $\sqrt{2}\times \mathrm{SEM}$[26]. The 95 percent confidence interval for the SEM (SEM_95_) and MDC (MDC_95_) was calculated by multiplying by the z-score of 1.96. LoA was calculated according to the Bland-Altman method [27] and a LoA plot was made for visual judgement. A paired t-test was performed to determine the systematic difference between the DASH scores at test and retest.

#### Construct validity

Construct validity was evaluated by testing six a priori hypotheses for the Pearson’s correlation coefficient between the DASH and the SPADI, SF-36 and the NPRS. A priori hypotheses were made based on the conceptual model of the measures and results of previous studies. As suggested by Rowntree [28] correlation coefficients below 0.2 were considered as very weak or negligible, between 0.2 and 0.4 as weak or low, between 0.4 and 0.7 as moderate, between 0.7 and 0.9 as strong, high or marked, and above 0.9 as very strong or very high. We hypothesized a high and positive correlation (0.7 to 0.9) between the DASH total score and the SPADI total score. We expected a negative, moderate correlation (-0.4 to -0.7) with the PF, BP, and PCS score of the SF-36. Furthermore, we hypothesized a negative, moderate correlation with the SF of the SF-36. The DASH score was expected to correlate higher with the PCS of the SF-36 than with the MCS.

## Results

Ninety-four patients met the inclusion criteria, of whom 29 were unwilling or unable to participate in the study and two were excluded because of generalized pain. Sixty-three patients (30 women, 33 men), with a mean age of 53 (Standard deviation [SD] 12.9) years, were included in the study. The mean duration of symptoms was 46.6 (SD 72.3) months, ranging from 2 to 420 months. Descriptive characteristics of the patients are shown in Table 1. Mean interval between the first and the second visit was 7.5 (SD 2.1) days, ranging from 7 to 21 days. Four missing values were found in both the DASH and SPADI at retest (completion rate, 93.7%) and these subjects were excluded in the analyses for test-retest reliability and calculation of measurement error. Two missing values (RP and MH) were found in the SF-36 (completion rate, 99.9%). The SF-36 records were scored with the QualityMetric’s missing data estimation (MDE). The DASH scores were considered to be normally distributed. No floor or ceiling effects were found. The scores ranged from 8.3 to 58.6 at the first visit and from 5.0 to 58.6 at the second visit.

**Table 1:** Descriptive characteristics of the patients n = 63

**Variable**	**No. (%) or mean ± SD**
Age, years	53.3 ± 12.9
Women	30 (48)
Men	33 (52)
Symptom duration, months	46.6 ± 72.3
Affected shoulder	
Right	19 (48)
Left	30 (30)
Bilateral	14 (22)
Working/student full time	29 (46)
Sick listed 100%	8 (13)
Partial sick listed	9 (14)
Retired	14 (22)
Receiving disability benefit	2 (3)
Unemployed	1 (2)

### Reliability

**Figure 1:**
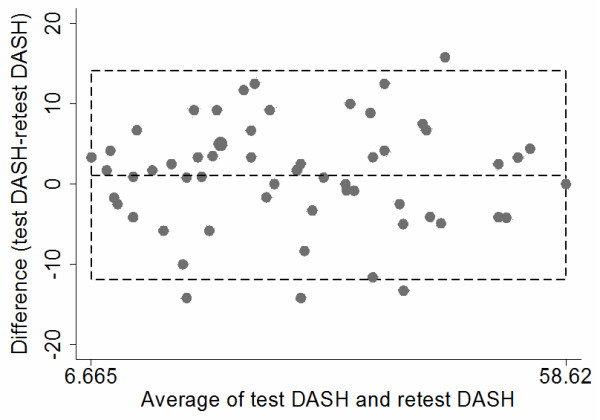
**Limits of agreement plot n = 59.** The difference in scores between DASH test and retest DASH plotted against the average scores of both test occasions. The middle dotted line represents mean difference (1.1). The bottom and top dotted line represent the 95% limits of agreement (-11.9, 14.1). Four out of 59 (6.8%) were outside the limits of agreement.

Reliability data are presented in Table 2. The internal consistency estimate (n = 63) of Cronbach’s alpha was 0.93. Item-to-total correlations ranged from 0.36 to 0.81. The DASH mean score (n = 59) at the first visit was 29.5 (SD 14.0) and at the second visit 28.4 (SD 14.0), giving a mean difference of 1.1 (SD 6.64) (95% confidence interval [CI] -0.65 to 2.82). ICC (n = 59) was 0.89 (95% CI 0.82 to 0.93). The SEM was 4.7 and SEM_95_ was 8.3. The MDC was 6.7 and MDC_95_ was 13.1. The 95% LoA was calculated to be between -11.9 and 14.1, and 4 out of 59 (6.8%) were outside the LoA (Figure 1).

**Table 2:** **Cronbach’s alpha, item-to-total correlations, intraclass correlation coefficient (ICC), standard error of measurement (SEM) and minimally detectable change (MDC**_**95**_**) of the Norwegian version of the DASH**

**Cronbach’s alpha**	**Item-to-total correlations**	**ICC (95% CI)**	**SEM**	**MDC**_**95**_
0.93	0.36-0.81	0.89 (0.82-0.93)	4.7	13.1

### Construct validity

The statistical analysis of correlation coefficients between DASH and the SPADI, SF-36 and NPRS are shown in Table 3. We found a high positive correlation between the DASH and SPADI (0.75). The DASH showed a moderate negative correlation with the PF (-0.48), BP (-0.62), and PCS (-0.59) of the SF-36, and a moderate positive correlation with the NPRS (0.58). The DASH correlated higher with the PCS (-0.59) than with the MCS (-0.17) score. It had a low negative correlation to the SF (-0.35) of the SF-36. All correlations were in line with a priori hypotheses except the low negative correlation to the SF of the SF-36, which mean that eighty-three percent of the hypotheses of correlation were confirmed.

**Table 3:** Descriptive statistics of the DASH, SPADI, SF-36 and NPRS at test and Pearson’s correlation coefficients for DASH n = 63

			**Correlations**	
	**Mean**	**SD**	**DASH**	**p-value**
The Disabilities of the Arm, Shoulder and Hand questionnaire (DASH)	29.4	13.8	-	-
The Shoulder Pain and Disability Index (SPADI)	36.2	16.6	0.75	<0.001
Numeric Pain Rating Scale (NPRS)	4.1	2.0	0.58	<0.001
The Short Form 36 Health Survey (SF-36)				
Physical functioning	77.9	14.8	-0.48	<0.001
Role-physical	35.3	37.7	-0.49	<0.001
Bodily pain	44.3	15.3	-0.62	<0.001
General health	73.3	20.2	-0.30	0.018
Vitality	54.1	21.5	-0.41	0.001
Social functioning	84.9	18.7	-0.35	0.005
Role-emotional	70.4	39.8	-0.25	0.051
Mental health	78.8	15.2	-0.28	0.029
Physical component summary	38.3	7.0	-0.59	<0.001
Mental component summary	50.9	10.1	-0.17	0.179

## Discussion

Our results provide evidence for good reliability and validity of the Norwegian language version of the DASH in patients with shoulder impingement syndrome. The results are comparable to those reported for the original English version and other language versions.

### Reliability

The Cronbach’s alpha coefficient of 0.93 indicated a good internal consistency and is similar to previous reported values. In the original version [5] and other languages versions [29-31] the reported Cronbach’s alpha was 0.96. A value of 0.93 was also reported for the Norwegian language version in patients with rheumatic diseases [32]. A Cronbach’s alpha between 0.70 and 0.95 have been proposed as a measure of good internal consistency [22].

Internal consistency assessed by item-to-total correlations ranged from 0.36 to 0.81.The item-to-total correlations were above the threshold value of 0.3, suggesting that the correlation between each item and the total score of the questionnaire were acceptable. Item-to-total correlations reported for the original English version of the DASH ranged from 0.49 to 0.87 [33]. Values reported for other language versions of the DASH ranged from 0.27-0.88 [30,34,35].

The test-retest reliability of the DASH was calculated to 0.89, which is considered to be excellent [24]. Studies for other languages versions have also shown high test-retest reliability with ICC values varying from 0.82 to 0.96, [30,36-42]. We retested the patients after approximately one week, which is within the recommended time frame ranging from two days to two weeks [43]. Due to this short time interval, most of the patients reported their shoulder pain as unchanged at the second visit.

In order to detect any systematic changes, the mean difference between the DASH test and retest was visualized in a limits of agreement plot. The limits of agreement plot may reveal systematic changes between the difference and the average of the DASH or outlying observations. Four out of 59 (6.8%) observations exceeded the limits of agreement. The mean difference between DASH test and retest was 1.1 (95% CI -0.65 to 2.82) and showed no systematic change. There was no apparent tendency for the mean difference to vary systematically with the average score.

The SEM was 4.7 points, SEM_95_ was 8.3 and the MDC_95_ was 13.1. These results correspond well with the measurement error values reported for the original English version with a SEM of 4.6 points and a MDC_95_ of 12.8 points [40]. The interpretation of SEM_95_ is that if a patient has a measured DASH score of for example 50 points at an initial test, the clinician can be 95 percent confident that the patient’s true score lies somewhere between 42 and 58 DASH points. The MDC_95_ of 13.1 indicates that the clinician can be 95 percent confident that a change has occured if the measured DASH score at retest has changed more than 13.1 points.

A distinction between MDC and minimally important change (MIC) is useful when interpreting change scores in PROMs [26]. The MDC is a measure of the statistically important change. The MIC can be defined as the smallest change in score which is perceived as important by patients, clinicians, or relevant others [26,44]. Different methods may be used to estimate this threshold value which indicates if a patient is better or worse [45]. A change above 15 points is found to be above most estimates of MIC for the DASH, and is considered to be the most accurate change score for discriminating between improved and unimproved patients [5].

### Construct validity

Our results of construct validity agree with previous studies [5]. The expected high positive correlation between DASH and SPADI was confirmed with a correlation coefficient of 0.75. Both the DASH and SPADI intend to measure activity limitations and pain (symptoms). However, there are differences in the content of these questionnaires. The DASH is found to be more wide-ranging than the SPADI and can be linked to 23 categories of the International Classification of Functioning, Disability and Health model (ICF), whereas SPADI is linked to six categories [46].

We had hypothesized a moderate and negative correlation with the Social Functioning domain of the SF-36, because the DASH is also meant to measure components of the social dimension: family care, occupational and socializing with friends and relatives. A moderate correlation with the Social Functioning of SF-36 has been reported in several other languages versions with correlation coefficients ranging from -0.53 to -0.64 [31,36,47-49]. The expected moderate negative correlation with the SF subscale of SF-36 was not confirmed. The low negative correlation to the SF (-0.35) may indicate that the Norwegian language version of DASH to a limited degree identifies the social dimension of functional status in this population, as measured by the SF-36.

### DASH scores

The DASH questionnaire measures whether the respondent has the capacity to do an activity, regardless of how it is performed. It is scored from 0 (best) to 100 (worst). A mean DASH score of 10 have been reported for the general population of the United States [33]. A mean score of 13 have been reported for both the general population in Norway [50] and a working population in Germany [51]. A Norwegian study of physical function in adult acquired major upper-limb amputees reported a mean DASH score of 22.7 [52]. The mean score of 29.4 (SD ± 13.8) in our study population indicated a more severe level of disability compared with these populations. The level of disability in our study population is comparable to other studies of patients with shoulder impingement syndrome [53,54].

### Study limitations

The DASH was designed to measure physical function and symptoms in patients with musculoskeletal disorders of the upper extremity. The results in this study are limited to patients with the primary diagnosis of shoulder impingement syndrome and can not be generalized to other disorders of the upper extremity. Another limitation of this study is that we did not evaluate responsiveness, which has been defined as the ability of a questionnaire to detect change over time in the construct to be measured [55]. Responsiveness is considered as an important measurement property of a PROM used for treatment evaluation and needs to be evaluated for the Norwegian version in future research.

## Conclusions

This study demonstrated excellent test-retest reliability, good internal consistency and established error values for the Norwegian language version of the DASH. Furthermore, this study provided evidence supporting the DASH as a valid measure of physical disability and symptoms in patients with shoulder impingement syndrome.

## Competing interests

The authors declare that they have no competing interests.

## Authors’ contributions

IS and BH obtained funding for the study. All authors participated in planning and design of the study. IS and BH collected and analyzed the data. BH drafted the manuscript. All authors contributed to interpretation of the study results and participated in revisions of the manuscript. All authors read and approved the final manuscript.

## Pre-publication history

The pre-publication history for this paper can be accessed here:

http://www.biomedcentral.com/1471-2474/15/78/prepub

## References

[B1] Bot SDM Terwee CB van der Windt DAWN Bouter LM Dekker J de Vet HCW Clinimetric evaluation of shoulder disability questionnaires: a systematic review of the literature Ann Rheum Dis 2004 63 335 341 10.1136/ard.2003.007724 15020324 PMC1754942

[B2] Angst F Schwyzer HK Aeschlimann A Simmen BR Goldhahn J Measures of adult shoulder function: Disabilities of the Arm, Shoulder, and Hand Questionnaire (DASH) and its short version (QuickDASH), Shoulder Pain and Disability Index (SPADI), American Shoulder and Elbow Surgeons (ASES) Society standardized shoulder assessment form, Constant (Murley) Score (CS), Simple Shoulder Test (SST), Oxford Shoulder Score (OSS), Shoulder Disability Questionnaire (SDQ), and Western Ontario Shoulder Instability Index (WOSI) Arthritis Care Res 2011 63 Suppl 11 S174 S188 10.1002/acr.20630 22588743

[B3] Hudak PL Amadio PC Bombardier C Beaton D Cole D Davies A Hawker G Katz JN Makela M Marx RG Punnett L Wright J Development of an upper extremity outcome measure: The DASH (disabilities of the arm, shoulder and hand) Am J Ind Med 1996 29 602 608 10.1002/(SICI)1097-0274(199606)29:6<602::AID-AJIM4>3.0.CO;2-L 8773720

[B4] Beaton DE Davis AM Hudak P McConnell S The DASH (Disabilities of the Arm, Shoulder and Hand) Outcome Measure: What do we know about it now? Br J Hand Ther 2001 6 109 118

[B5] Kennedy CA Beaton DE Solway S McConnell S Bombardier C The DASH and QuickDASH Outcome Measure User’s Manual 2011 3 Toronto, Ontario: Institute for Work & Health

[B6] The DASH outcome measure website http://www.dash.iwh.on.ca/

[B7] Roy JS MacDermid JC Woodhouse LJ Measuring shoulder function: a systematic review of four questionnaires Arthritis Care Res 2009 61 623 632 10.1002/art.24396 19405008

[B8] Van der Windt DAWM Koes BW de Jong BA Bouter LM Shoulder disorders in general practice: incidence, patient characteristics, and management Ann Rheum Dis 1995 54 959 964 10.1136/ard.54.12.959 8546527 PMC1010060

[B9] Silva L Andréu JL Muñoz P Pastrana M Millán I Sanz J Barbadillo C Fernández-Castro M Accuracy of physical examination in subacromial impingement syndrome Rheumatology (Oxford) 2008 47 679 683 10.1093/rheumatology/ken101 18375403

[B10] Bigliani LU Levine WN Current concepts review. Subacromial impingement syndrome J Bone Joint Surg 1997 79 1854 1868 9409800

[B11] Michener LA McClure PW Karduna AR Anatomical and biomechanical mechanisms of subacromial impingement syndrome Clin Biomech (Bristol, Avon) 2003 18 369 379 10.1016/S0268-0033(03)00047-0 12763431

[B12] McKenna SP Doward LC The translation and cultural adaptation of patient-reported outcome measures Value Health 2005 8 89 91 10.1111/j.1524-4733.2005.08203.x 15804316

[B13] Hawkins RJ Kennedy JC Impingement syndrome in athletes Am J Sports Med 1980 8 151 158 10.1177/036354658000800302 7377445

[B14] Finsen V Norwegian version of the DASH questionnaire for evaluation of the arm, shoulder and hand Tidsskr Nor Laegeforen 2008 128 1070 (In Norwegian) 18451890

[B15] Roach KE Budiman-Mak E Songsiridej N Lertratanakul Y Development of a shoulder pain and disability index Arthritis Care Res 1991 4 143 149 10.1002/art.1790040403 11188601

[B16] Tveitå EK Ekeberg OM Juel NG Bautz-Holter E Responsiveness of the shoulder pain and disability index in patients with adhesive capsulitis BMC Musculoskelet Disord 2008 9 161 10.1186/1471-2474-9-161 19055757 PMC2633286

[B17] Ekeberg OM Bautz-Holter E Tveitå EK Keller A Juel NG Brox JI Agreement, reliability and validity in 3 shoulder questionnaires in patients with rotator cuff disease BMC Musculoskelet Disord 2008 9 68 10.1186/1471-2474-9-68 18482438 PMC2409321

[B18] SF-36.org http://www.sf-36.org/

[B19] Loge JH Kaasa S Hjermstad MJ Kvien TK Translation and performance of the Norwegian SF-36 health survey in patients with rheumatoid arthritis. I. data quality, scaling assumptions, reliability, and construct validity J Clin Epidemiol 1998 51 1069 1076 10.1016/S0895-4356(98)00098-5 9817124

[B20] Downie WW Leatham PA Rhind VM Wright V Branco JA Anderson JA Studies with pain rating scales Ann Rheum Dis 1978 37 378 381 10.1136/ard.37.4.378 686873 PMC1000250

[B21] Mintken PE Glynn P Cleland JA Psychometric properties of the shortened disabilities of the Arm, Shoulder, and Hand Questionnaire (QuickDASH) and Numeric Pain Rating Scale in patients with shoulder pain J Shoulder Elbow Surg 2009 18 920 926 10.1016/j.jse.2008.12.015 19297202

[B22] Terwee CB Bot SDM de Boer MR van der Windt DAWN Knol DL Dekker J Bouter LM de Vet HCW Quality criteria were proposed for measurement properties of health status questionnaires J Clin Epidemiol 2007 60 34 42 10.1016/j.jclinepi.2006.03.012 17161752

[B23] Field AP Discovering statistics using SPSS 2009 Los Angeles, [Calif.]; London: SAGE

[B24] Fleiss JL The Design and Analysis of Clinical Experiments 1986 New York: Wiley

[B25] Weir JP Quantifying test-retest reliability using the intraclass correlation coefficient and the SEM J Strength Cond Res 2005 19 231 15705040 10.1519/15184.1

[B26] de Vet HC Terwee CB Ostelo RW Beckerman H Knol DL Bouter LM Minimal changes in health status questionnaires: distinction between minimally detectable change and minimally important change Health Qual Life Outcomes 2006 4 54 10.1186/1477-7525-4-54 16925807 PMC1560110

[B27] Bland JM Altman DG Measuring agreement in method comparison studies Stat Meth Med Res 1999 8 135 160 10.1191/096228099673819272 10501650

[B28] Rowntree D Statistics without tears: a primer for non-mathematicians 2004 Boston: Allyn and Bacon

[B29] Offenbächer M Ewert T Sangha O Stucki G Validation of a German version of the Disabilities of Arm, Shoulder and Hand questionnaire (DASH-G) Z Rheumatol 2003 62 168 177 10.1007/s00393-003-0461-7 12721705

[B30] Atroshi I Gummesson C Andersson B Dahlgren E Johansson A The disabilities of the arm, shoulder and hand (DASH) outcome questionnaire Acta Orthop Scand 2000 71 613 618 10.1080/000164700317362262 11145390

[B31] Themistocleous GS Goudelis G Kyrou I Chloros GD Krokos A Galanos A Gerostathopoulos NE Soucacos PN Translation into Greek, cross-cultural adaptation and validation of the Disabilities of the Arm, Shoulder, and Hand Questionnaire (DASH) J Hand Ther 2006 19 350 357 10.1197/j.jht.2006.04.014 16861133

[B32] Christie A Hagen KB Mowinckel P Dagfinrud H Methodological properties of six shoulder disability measures in patients with rheumatic diseases referred for shoulder surgery J Shoulder Elbow Surg 2009 18 89 95 10.1016/j.jse.2008.07.008 19095181

[B33] Hunsaker FG Cioffi DA Amadio PC Wright JG Caughlin B The American Academy of Orthopaedic Surgeons outcomes instruments: normative values from the general population J Bone Joint Surg 2002 84 208 215 11861726 10.2106/00004623-200202000-00007

[B34] Durand MJ Vachon B Hong QN Loisel P The cross-cultural adaptation of the DASH questionnaire in Canadian French J Hand Ther 2005 18 34 39 10.1197/j.jht.2004.10.010 15674785

[B35] Lee EWC Lau JSY Chung MMH Li APS Lo SK Evaluation of the Chinese version of the Disability of the Arm, Shoulder and Hand (DASH-HKPWH): cross-cultural adaptation process, internal consistency and reliability study J Hand Ther 2004 17 417 423 15538683

[B36] Padua R Padua L Ceccarelli E Romanini E Zanoli G Amadio PC Campi A Italian version of the Disability of the Arm, Shoulder and Hand (DASH) questionnaire. Cross-cultural adaptation and validation J Hand Surg Br 2003 28 179 186 10.1016/S0266-7681(02)00303-0 12631494

[B37] Imaeda T Toh S Nakao Y Nishida J Hirata H Ijichi M Kohri C Nagano A Validation of the Japanese Society for Surgery of the Hand version of the Disability of the Arm, Shoulder, and Hand questionnaire J Orthop Sci 2005 10 353 359 10.1007/s00776-005-0917-5 16075166 PMC2780667

[B38] Fayad F Lefevre-Colau MM Macé Y Fermanian J Mayoux-Benhamou A Roren A Rannou F Roby-Brami A Gautheron V Revel M Poiraudeau S Validation of the French version of the Disability of the Arm, Shoulder and Hand questionnaire (F-DASH) Joint Bone Spine 2008 75 195 200 10.1016/j.jbspin.2007.04.023 17983829

[B39] Mousavi SJ Parnianpour M Abedi M Askary-Ashtiani A Karimi A Khorsandi A Mehdian H Cultural adaptation and validation of the Persian version of the Disabilities of the Arm, Shoulder and Hand (DASH) outcome measure Clin Rehabil 2008 22 749 757 10.1177/0269215508085821 18678575

[B40] Beaton DE Katz JN Fossel AH Wright JG Tarasuk V Bombardier C Measuring the whole or the parts? Validity, reliability, and responsiveness of the Disabilities of the Arm, Shoulder and Hand outcome measure in different regions of the upper extremity J Hand Ther 2001 14 128 142 10.1016/S0894-1130(01)80043-0 11382253

[B41] Orfale AG Araújo PMP Ferraz MB Natour J Translation into Brazilian Portuguese, cultural adaptation and evaluation of the reliability of the disabilities of the arm, shoulder and hand questionnaire Braz J Med Biol Res 2005 38 293 10.1590/S0100-879X2005000200018 15785841

[B42] Slobogean GP Noonan VK O’Brien PJ The reliability and validity of the disabilities of arm, shoulder, and hand, EuroQol-5D, health utilities index, and Short Form-6D outcome instruments in patients with proximal humeral fractures J Shoulder Elbow Surg 2010 19 342 348 10.1016/j.jse.2009.10.021 20189839

[B43] Marx RG Menezes A Horovitz L Jones EC Warren RF A comparison of two time intervals for test-retest reliability of health status instruments J Clin Epidemiol 2003 56 730 735 10.1016/S0895-4356(03)00084-2 12954464

[B44] de Vet HCW The minimal detectable change should not replace the minimal important difference J Clin Epidemiol 2010 63 804 805 10.1016/j.jclinepi.2009.12.015 20399609

[B45] Beaton DE van Eerd D Smith P van der Velde G Cullen K Kennedy CA Hogg-Johnson S Minimal change is sensitive, less specific to recovery: a diagnostic testing approach to interpretability J Clin Epidemiol 2011 64 487 496 10.1016/j.jclinepi.2010.07.012 21109396

[B46] Roe Y Soberg HL Bautz-Holter E Ostensjo S A systematic review of measures of shoulder pain and functioning using the International classification of functioning, disability and health (ICF) BMC Musculoskelet Disord 2013 14 73 10.1186/1471-2474-14-73 23445557 PMC3668165

[B47] SooHoo NF McDonald AP Seiler JG McGillivary GR Evaluation of the construct validity of the DASH questionnaire by correlation to the SF-36 J Hand Surg Am 2002 27 537 541 10.1053/jhsu.2002.32964 12015732

[B48] Raven EEJ Haverkamp D Sierevelt IN van Montfoort DO Pöll RG Blankevoort L Tak PP Construct validity and reliability of the disability of arm, shoulder and hand questionnaire for upper extremity complaints in rheumatoid arthritis J Rheumatol 2008 35 2334 2338 10.3899/jrheum.080067 19004045

[B49] Lee EWC Chung MMH Li APS Lo SK Construct validity of the Chinese version of the disabilities of the arm, shoulder and hand questionnaire (DASH-HKPWH) J Hand Surg Br 2005 30 29 34 10.1016/j.jhsb.2004.09.010 15620488

[B50] Aasheim T Finsen V The DASH and the QuickDASH instruments. Normative values in the general population in Norway J Hand Surg Eur Vol 2014 39 140 144 10.1177/1753193413481302 23520389

[B51] Jester A Harth A Germann G Measuring levels of upper-extremity disability in employed adults using the DASH Questionnaire J Hand Surg Am 2005 30 1074 e1-1074.e10 16182070 10.1016/j.jhsa.2005.04.009

[B52] Ostlie K Franklin RJ Skjeldal OH Skrondal A Magnus P Assessing physical function in adult acquired major upper-limb amputees by combining the Disabilities of the Arm, Shoulder and Hand (DASH) Outcome Questionnaire and clinical examination Arch Phys Med Rehabil 2011 92 1636 1645 10.1016/j.apmr.2011.04.019 21872841

[B53] de Witte PB Henseler JF Nagels J Vliet Vlieland TP Nelissen RG The Western Ontario rotator cuff index in rotator cuff disease patients: a comprehensive reliability and responsiveness validation study Am J Sports Med 2012 40 1611 1619 10.1177/0363546512446591 22582227

[B54] Roy JS Moffet H McFadyen BJ Upper limb motor strategies in persons with and without shoulder impingement syndrome across different speeds of movement Clin Biomech (Bristol, Avon) 2008 23 1227 1236 10.1016/j.clinbiomech.2008.07.009 18757123

[B55] Mokkink LB Terwee CB Patrick DL Alonso J Stratford PW Knol DL Bouter LM de Vet HC The COSMIN study reached international consensus on taxonomy, terminology, and definitions of measurement properties for health-related patient-reported outcomes J Clin Epidemiol 2010 63 737 745 10.1016/j.jclinepi.2010.02.006 20494804

